# Lower Limb Myxofibrosarcoma Presenting as a Pulmonary Tumour Embolism: A Case Report

**DOI:** 10.7759/cureus.84535

**Published:** 2025-05-21

**Authors:** Hritik J John, Samuel J White, Pallavi Byrapu, Smita Raju

**Affiliations:** 1 Faculty of Health and Medical Sciences, University of Adelaide, Adelaide, AUS; 2 Department of Radiology, Royal Adelaide Hospital, Adelaide, AUS

**Keywords:** clinical case report, imaging findings, malignant myxofibrosarcoma, pulmonary embolism despite anticoagulation, pulmonary tumour embolism

## Abstract

Pulmonary tumour embolism is a rare complication of malignancy and should be considered a differential diagnosis if imaging features are atypical for a bland thrombus. It can arise from a range of primary tumours, including soft tissue sarcomas; however, reported cases are sparse. We present a case of pulmonary tumour embolism secondary to a large right knee myxofibrosarcoma, with diagnosis confirmed following biopsy of a pulmonary nodule and a palpable inguinal lymph node.

## Introduction

Pulmonary tumour embolism (PTE) typically originates from primary malignancies of the lung, colon, breast, and stomach, with few reported cases of PTE arising from soft tissue sarcomas [1]. Myxofibrosarcoma is one of the most common soft tissue sarcomas. It primarily arises in the extremities and typically affects individuals within their sixth to eighth decade of life with a slight male predominance [2]. The occurrence of PTE secondary to myxofibrosarcoma is exceptionally rare, with very few cases being described in the literature. Here, we report a rare and unusual case of lower limb myxofibrosarcoma presenting as PTE, highlighting several key learning points.

## Case presentation

A female in her mid-70s presented to the emergency department dyspneic after a choking episode. Examination revealed a new 2 L oxygen requirement and bibasal lung crepitations. Relevant background included bilateral unprovoked pulmonary embolism (PE) 10 years prior, treated with lifelong dabigatran, and a prior total knee replacement requiring a single-stage revision four months earlier.

**Figure 1:**
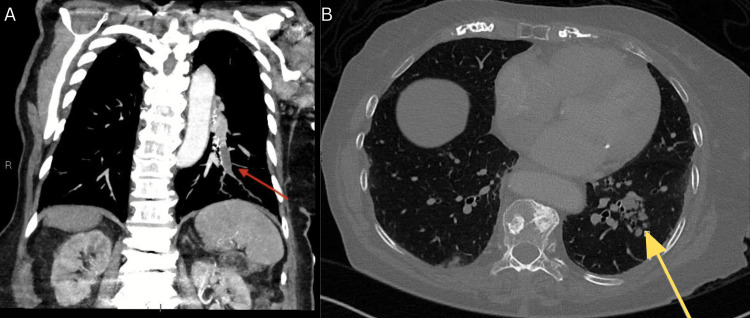
Initial CT of the chest post-contrast phase: (A) coronal maximum intensity projection view in soft tissue windows and (B) axial view. Coronal CT image demonstrates an occlusive filling defect in the left lateral basal segmental pulmonary artery with a distended lumen (red arrow). Axial CT image showing a cluster of nodules with a tree-in-bud pattern evident in the lateral basal segment of the left lower lobe (yellow arrow).

Initial non-dedicated post-contrast CT chest (Figure 1) revealed no endobronchial foreign body; however, it revealed left lateral basal and lingular segmental artery vessel filling defects, keeping with acute PE. Additional findings included tree-in-bud nodularities in bilateral lower lobes and a new 9 mm right upper lobe nodule. The working diagnosis was a recurrence of PE while on dabigatran. The patient was admitted and anticoagulated with therapeutic enoxaparin, which was transitioned to oral apixaban on discharge once symptoms improved. Vasculitic and thrombophilic blood panels were unremarkable, and recent outpatient mammogram, cervical screening, colonoscopy, and gastroscopy were all negative.

**Figure 2:**
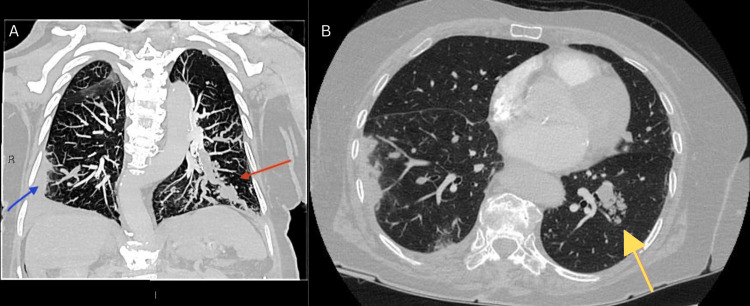
CT pulmonary angiogram completed three months after the initial presentation: (A) maximum intensity projection coronal view and (B) axial view. Coronal CT image demonstrates an occlusive tumour in the lateral basal segmental pulmonary artery with an irregular beading appearance, branching centrilobular nodules, and persisting luminal distension (red arrow). A peripheral lung infarct can also be seen in the right lower lobe (blue arrow). Axial CT image demonstrates persisting tree-in-bud pattern evident in in the lateral basal segment of the left lower lobe (yellow arrow).

The patient presented several weeks later with worsening dyspnoea and new right knee swelling. CT pulmonary angiogram (Figure 2) at the time revealed further luminal expansion of the lateral basal and lingular pulmonary arteries with filling defects and similar new imaging appearances in the right lower lobe despite anticoagulation. Pulmonary nodules were now innumerable, primarily perivascularly distributed, giving a diffuse tree-in-bud appearance, and the previous right upper lobe nodule increased in size from 9 mm to 18 mm. This unexpected evolution of pulmonary vascular and parenchymal change of uncertain aetiology prompted consideration of PTE as a differential diagnosis.

**Figure 3:**
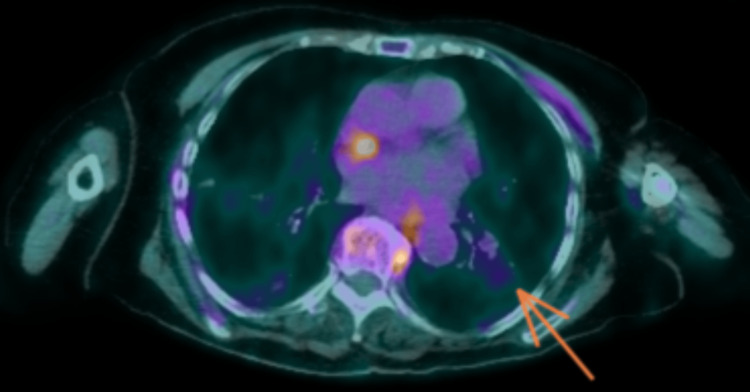
Axial section of fludeoxyglucose (18F) (FDG) positron emission tomography. The image demonstrates mild FDG activity of the thrombus in the lateral basal segmental branch of the left lower lobe (orange arrow).

**Figure 4:**
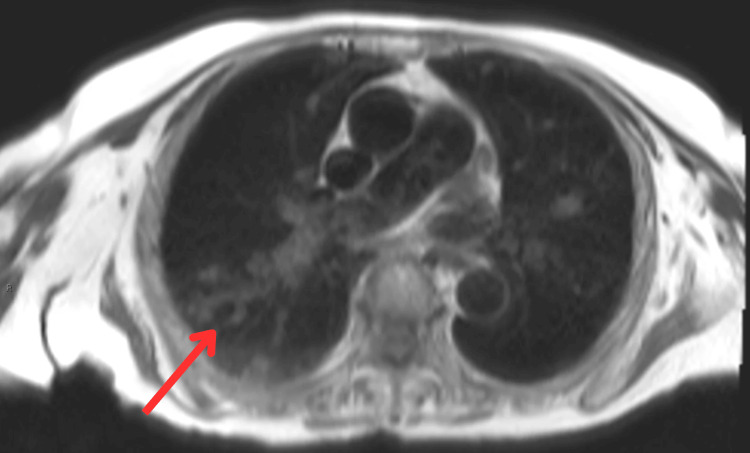
Cardiac MRI: axial T1 image at the level of the main pulmonary artery. The image demonstrates T1 intermediate-signal intense intraluminal lesions in the right pulmonary artery and posterior segmental pulmonary artery of the right upper lobe (red arrow).

CT-guided core biopsy of the growing right upper lobe nodule suggested myxomatous tissue. A fludeoxyglucose (18F) positron emission tomography (FDG-PET) scan (Figure 3) demonstrated low FDG avidity within the interatrial septum, minimally FDG-avid bilateral PE, and no FDG-avid primary lesion. Critically, the lower limbs were outside the field of view. Due to concern for intracardiac myxoma, the patient underwent cardiac MRI (Figure 4) and transoesophageal echocardiogram; however, no intracardiac mass was identified.

**Figure 5:**
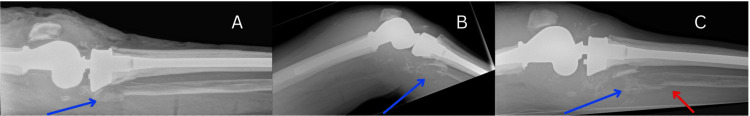
Lateral right knee radiographs: (A) day two post after the right knee replacement, (B) re-presentation seven months post-surgery with a new right knee swelling, and (C) most recent radiograph nine months post-surgery. The image demonstrates progressive ill-defined lytic change at the proximal fibula (blue arrow) across serial right knee radiographs over time. The most recent radiograph demonstrates a transverse fracture at the proximal shaft, compatible with pathological fracture (red arrow), periosteal reaction reminiscent of an aggressive process, and a large high density knee joint effusion/haemarthrosis.

**Figure 6:**
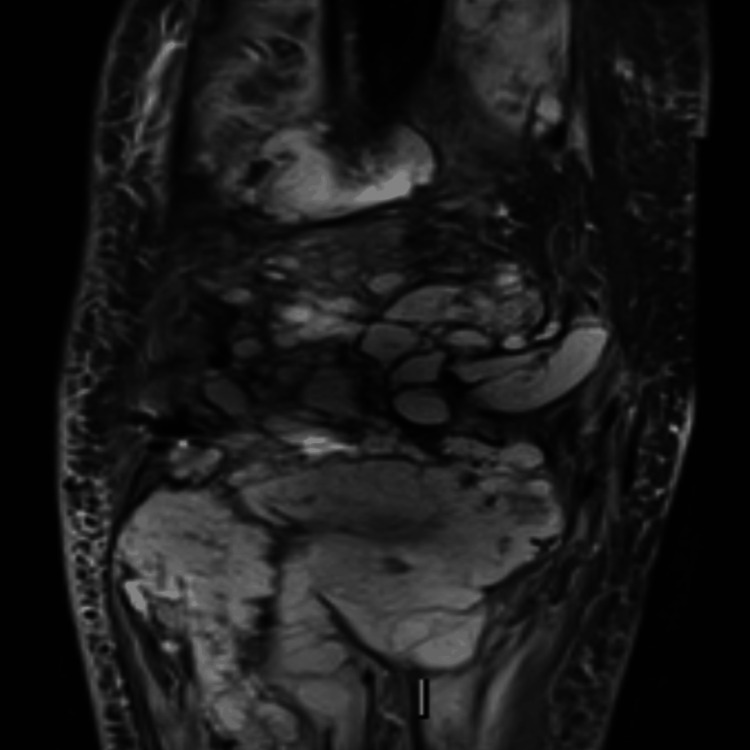
Coronal T1 fat-suppressed MRI of the right knee. The image demonstrates a large soft tissue mass involving the bones and soft tissue of the right knee. Artefacts from the prosthesis can be noted.

To investigate the right knee swelling, duplex venous ultrasound of the right lower limb showed no thrombus, and a right knee X-ray (Figure 5B) revealed resorption of the fibular head, stable since postoperative imaging (Figure 5A) several months prior. Orthopaedic assessment at the time attributed the knee swelling to likely spontaneous haemarthrosis secondary to anticoagulation. The patient soon re-presented with worsening dyspnoea and right knee swelling along with a new palpable right inguinal lymph node. A biopsy of this lymph node suggested myxofibrosarcoma. Repeat right knee X-ray (Figure 5C) demonstrated significant interval progression of fibular head osteolysis and a new acute pathological fracture of the fibular shaft. Right knee MRI (Figure 6) demonstrated a large lobulated circumferential periprosthetic mass, keeping with a primary myxofibrosarcoma.

**Figure 7:**
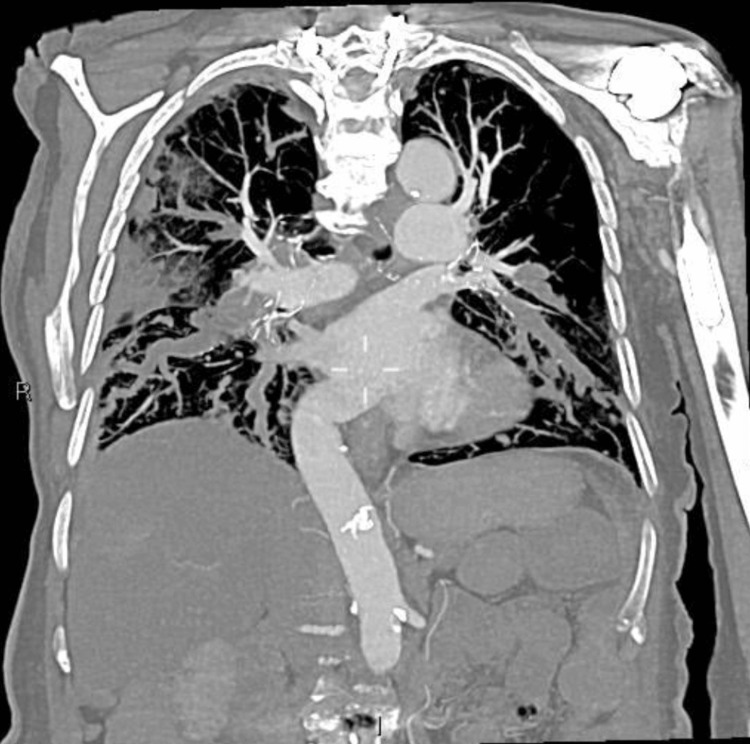
Coronal CT of the chest from staging chest-abdomino-pelvic CT six months after the initial presentation. The image demonstrates progressive bilateral tumour thrombi in pulmonary arterial branches with luminal distension.

Staging chest-abdominal-pelvic CT (Figure 7) demonstrated further progression of bilateral PE burden, innumerable enlarging pulmonary metastases, as well as mediastinal and right inguinal lymphadenopathy. The patient’s dyspnoea had progressively worsened over her several presentations, severely impacting her quality of life. After extensive multidisciplinary discussions, due to the burden of disease and symptoms, the patient opted for palliation and passed away three months after the initial presentation.

## Discussion

PTE is an exceedingly rare primary presentation of myxofibrosarcoma, with only one other case previously reported in an acute postoperative setting [1]. PE that persists or evolves unexpectedly despite anticoagulation should prompt consideration of a broad range of differentials, including PTE. In cases where initial malignancy screen is unremarkable, consideration of less common primary sites such as musculoskeletal malignancies of the limbs is important.

Distinguishing a bland thrombus and PTE was a challenging aspect of this case. Features typically supporting PTE as opposed to a bland thrombus on contrast-enhanced CT include vessel expansion, beading appearance of the pulmonary vasculature, and enhancement of intravascular filling defects [3-5]. Additionally, emboli avidity may be noted on FDG-PET [6]. While expansion of occluded vessels and beading were present, other supportive features of PTE, such as thrombus enhancement and FDG avidity, were absent. This may be attributed to the small size, low grade of the PTE, and myxomatous nature of the primary tumour [3-5,7]. Furthermore, the patient’s prior history of unprovoked PE and lack of a known malignancy resulted in a low initial level of suspicion for PTE. A key feature that prompted further investigation for possible PTE was worsening embolic burden despite appropriate anticoagulation.

Another learning point is the broad differential diagnosis for centrilobular pulmonary nodules. Tree-in-bud pattern refers to a sign seen on thin-section chest CT appearing as several centrilobular nodules with a linear branching pattern, which can be indicative of either endo- or peri-bronchiolar disease. Most commonly, it is the result of a benign aetiology, particularly endobronchial spread of infection. Other causes include congenital, idiopathic, and immunological disorders, as well as aspiration events [8]. PTE is a rarely reported aetiology for this sign, arising due to translocation of tumour cells to centrilobular arteries or intimal fibrocellular hyperplasia of pulmonary arteries [8,9]. In cases where there is persistent centrilobular nodular change despite treatment or tree-in-bud sign associated with pulmonary embolism, the possibility of tumour embolism should be raised as a differential diagnosis.

## Conclusions

Lower limb myxofibrosarcoma presenting as PTE is an exceedingly rare phenomenon. Diagnosis can be challenging, especially in cases such as this of initial low clinical suspicion. However, the diagnosis does have significant implications for patient management and prognosis. This case highlights the importance of interrogating the PE burden that worsens despite treatment and considering uncommon primary sites when initial malignancy screens are unremarkable.
